# Differences in dietary pattern between obese and eutrophic children

**DOI:** 10.1186/1756-0500-4-567

**Published:** 2011-12-29

**Authors:** Emilia A Balthazar, Maria RM de Oliveira

**Affiliations:** 1 Food and Nutrition Department, School of Pharmaceutical Sciences at the São Paulo State University (UNESP), road Araraquara-Jau km1, CP 502, 14801-902, Araraquara, SP - Brasil; 2 Education department, Biosciences Institute, São Paulo State University (UNESP), District Rubião junior, CP 510, 18618-000, Botucatu, SP - Brasil

## Abstract

**Background:**

Excessive consumption of energy is a decisive factor of obesity, but a simple quantitative assessment of consumption between obese and eutrophic individuals not always explains the problem, raising questions about the importance of the qualitative aspects of food. Therefore, the purpose of this study was to evaluate the differences in nutrient composition and meal patterns between eutrophic and obese schoolchildren.

**Methods:**

The diet of 83 children (42 obese and 41 eutrophic), aged between 7 and 11 years of age, was assessed by two non-consecutive dietary recalls. After the software analysis of macro and micronutrients composition, the different types and amount of legumes, fruits and vegetables were analyzed to verify the dietary patterns.

**Results:**

No differences were verified in energy consumption between the groups (eutrophic = 1934.2 ± 672.7 kcal, obese = 1835.8 ± 621.2 kcal). In general, children showed consumption within the recommended ranges of carbohydrates, lipids and proteins. The average consumption of fiber was higher in the eutrophic group (20.7 g) when compared to the obese group (14.8 g). The dietary fiber was strongly correlated with the number of servings of beans (r = 0.77), when compared to fruits (r = 0.44) and leafy vegetables (r = 0.13). It was also observed that the higher the consumption of fiber and beans, the lower the proportion of dietary fat (r = -0.22) in the diet. Generally, there was a low consumption of fiber (20.7 g = eutrophic group/14.8 g = obese group), beans (1.1 portions in the eutrophic and obese groups), fruits (0.7 portions eutrophic group and 0.6 obese group) and vegetables (1.3 eutrophic group and 1.1 obese group).

**Conclusions:**

It is concluded that the obesity was more related to a dietary pattern of low intake of dietary fiber than excessive energy consumption and macronutrients imbalance.

## Background

Globally, children and adolescents are getting fatter and this situation seems irreversible [1-3]. A larger body size is not the main problem of obesity, but the numerous complications followed by it, such as, hypertension, dyslipidemia and type 2 diabetes [4].

Genetic, physiological and metabolic factors can influence the outcome of obesity. However, the factors that could explain the growing number of obese children appear to be more related to changes in lifestyle and eating habits [5,6].

An increased energy intake and changes in meal patterns, such as replacement of family meals with processed foods, may be important contributors to weight gain [7-9].

In different regions of Brazil, studies are indicating a change in meal pattern. According to the Brazilian household budget survey, from 2002/03 to 2008/09, the consumption of beans and rice decreased in the population, while there was an increase in the consumption of soft drinks, beer and mineral water [10].

The average annual *per capita *purchase decreased 40.5% for milled rice (24.5 kg to 14,6 kg) and 26.4% for beans (12.4 Kg to 9.1 kg) in this period. Fruits and vegetables accounted for only 2.8% of the total calories in the year of 2008 [10]. A greater exposure to processed foods with high levels of energy, saturated fat and cholesterol combined with a low intake of vegetables, fruits and legumes may in part contribute to the increase of overweight and obesity in adults and children.

Thus, this study aims to evaluate the differences in nutrient composition and the meal patterns between eutrophic and obese schoolchildren.

## Results

### Energy and macronutrients consumed by eutrophic and obese children

Regarding the macronutrients, proteins, carbohydrates and fat were analyzed as well as the energy consumption. No differences were observed in the consumption of macronutrients and energy between eutrophic and obese children (table 1). Most students, regardless of the group, consumed the macronutrients according to the Dietary Reference Intakes (DRIs) recommendation (table 1).

**Table 1:** Comparison of the adequacy of nutrient and energy consumption between the eutrophic and obese children

Energy and Nutrients	Children		
		
	Eutrophic (n = 41)	Obese (n = 42)	
	**Mean ± sd**	**Mean ± sd**	*p*-value*
*Energy consumption - kcal*	1934.2 ± 672.7	1835.8 ± 621.2	0.491
*Protein- g*	73.6 ± 30.1	73.1 ± 25.2	0.822
*Carbohydrate- g*	284.9 ± 93.6	270.3 ± 95.5	0.483
*Fat - g*	57.6 ± 29.2	52.6 ± 25.3	0.397
*Dietary fiber - g*	20.7 ± 11.4	14.8 ± 9.1	0.011
*Iron -mg*	15.7 ± 3.0	14.3 ± 2.7	0.243
*Vitamin A *- mcg	512.6 ± 316.3	917.2 ± 534.2	0.028
*Vitamin C - mg*	63.07 ± 56.3	64.4 ± 53.3	0.986
*Calcium -mg*	678.6 ± 295.9	613.8 ± 295.3	0.320
			
	n (%)	n (%)	
*Protein:*			
Low intake	5 (12.2)	1 (2.4)	ρ = 0.084 χ^2 ^= 3.000
Adequate intake	36 (87.8)	41 (97.6)	
High intake	0 (0.0)	0 (0.0)	
			
*Carbohydrate:*			
Low intake	1 (2.4)	0 (0.0)	ρ = 0.593 χ^2 ^= 1.047
Adequate intake	33 (80.5)	35 (83.3)	
High intake	7 (17.1)	7 (16.7)	
			
*Fat:*			
Low intake	19 (46.3)	22 (52.4)	ρ = 0.435 χ^2 ^= 0.805
Adequate intake	19 (46.3)	18 (42.9)	
High intake	3 (7.3)	2 (4.8)	
			
*Dietary fiber:*			
Low recommended	70.7(29)	92.9 (39)	ρ = 0.019 χ^2 ^= 5.447**
Recommended	29.3 (12)	7.1 (3)	
			
*Iron:*			
Inadequate intake	0 (< 1.0)	0 (< 1.0)	
Adequate intake	41 (> 99.0)	42 (> 99.0)	
*Vitamin A:*			
Inadequate intake	27 (66.0)	7 (16.0)	ρ < 0.0001 χ^2 ^= 20.756
Adequate intake	14 (34.0)	35 (84.0)	
			
*Vitamin C:*			
Inadequate intake	11 (27.0)	4(9.0)	ρ = 0.078 χ^2 ^= 3.109
Adequate intake	30 (73.0)	38 (91.0)	
			
*Calcium:*			
Low recommended	38 (92.7)	40 (95.0)	P = 0.625
Recommended	3(7.3)	2 (5.0)	χ^2 ^= 0.239**

*by student-t test between the eutrophic and obese children; **by Yates' chi-square test

### Dietary fiber and portions of food (beans, fruits, leafy vegetables and vegetables) consumed by eutrophic and obese children

The consumption of dietary fiber was statistically different between the groups, being higher in the eutrophic group (table 1). The correlations between the dietary fiber and BMI, waist circumference percent body fat were weak and negative, but statistically significant (table 2). It was verified that the number of bean servings (S. beans) presented a strong correlation with dietary fiber (g), whereas, the servings of fruits presented weak correlation and vegetables and leafy vegetables presented null correlation (table 3).

**Table 2:** Correlation between dietary fiber and anthropometric variables of the schoolchildren

	
Measures	Dietary fiber consumed per gram (n = 83)
BMI (kg/m^2^)	r (*p*-value)* -0.313 (0.004)
Waist Circumference (cm).	-0.281 (0.010)
Percentage of body fat	-0.296 (0.007)

*by Pearson correlation; BMI = body mass index

**Table 3:** Correlations between dietary fiber and consumed portions of beans, fruits and vegetables, according the children groups

	Dietary fiber (g)					
	
Servings	Total Sample (n = 83)		Eutrophic Children (n = 41)		Obese Children (n = 42)	
	r*	***p*-Value**	r*	***p*-Value**	r*	***p*-Value**

Beans	0.769	p < 0.0001	0.898	p < 0.0001	0.672	p < 0.0001
Fruits	0.418	p < 0.0001	0.484	0.001	0.299	0.054
Vegetable	0.059	0.596	0.233	0.142	-0.185	0.240
Leafy vegetable	0.126	0.255	0.19	0.246	0.02	0.924

*by Pearson correlation

Overall, in the eutrophic group the correlations of fiber with fruits and beans were stronger when compared to the obese group. In the eutrophic group, the correlation between fiber and beans was strong, while in the obesity group the correlation was medium. Students in the eutrophic group consumed an average of 1.1 S. beans, 0.7 serving of fruit and 1.3 servings of leafy vegetables and vegetables. The obese group consumed an average of 1.1 S. beans, 0.6 serving of fruit, 1.1 servings of leafy vegetables and vegetables. In the eutrophic group, the most consumed fruits were apple (41.9%), banana (16.0%), watermelon (15.2%), tangerine (11.2%) and other fruits (15.7%) and in the obesity group, they were apple (30.3%), banana (26.3%), orange (14.8%), Mango (7.3%) and other fruits (21.3%).

In table 4, the children were divided into tertiles according to the consumption of beans, in ascending order (from the lowest to the highest consumption of S. beans). In the total sample (obese + eutrophic children), as the intake of S. beans increased, the amount of fiber and iron also increased while the proportion of fat decreased.

**Table 4:** Amount of dietary fiber, iron and proportion of fat in the diet according to the amount of servings of beans consumed by eutrophic and obese children

	Variables		
	
	
Groups	Dietary Fiber (g)	Proportion of fat in diet (%)	Iron in diet (mg)
	**Mean ± sd**	**Mean ± sd**	**Mean ± sd**

*Eutrophic children (41)*			
First tertile S. Beans	9.9 ± 4.8^ad^	28.8 ± 6.7a	12.5 ± 4.2ac
Second tertile S. Beans	20.3 ± 5.7^b^	24.9 ± 4.3^b^	13.4 ± 3.3^a^
Third tertile S. Beans	31.3 ± 6.3^c^	22.0 ± 5.3^b^	20.2 ± 5.9^b^
*Obese children (42)*			
First tertile S. Beans	8.4 ± 3.0^a^	26.4 ± 6.3^ab^	11.6 ± 4.4^a^
Second tertile S. Beans	15.7 ± 7.5^bd^	26.1 ± 5.0^ab^	14.1 ± 4.5^ca^
Third tertile S. Beans	17.9 ± 6.6^b^	23.2 ± 5.9^b ^	17.0 ± 5.0^bc^
P - Value*	*p *< 0.00001	0.030	*p *< 0.00001

*Total sample (83)*			
First tertile S. Beans	9.3 ± 4.1^e^	27.6 ± 6.6^e^	12.1 ± 4.2^e^
Second tertile S. Beans	16.9 ± 6.9^f^	25.5 ± 4.5^e^	13.9 ± 4.8^e^
Third tertile S. Beans	24.4 ± 8.9^g^	22.6 ± 5.7^f^	18.4 ± 5.2^f^
P - Value*	*p *< 0.00001	0.010	*p *< 0.00001

* = by ANOVA between the eutrophic and obese children, same letters in the same column accompanying the values indicate that the values do not differ according to the Tukey test, after ANOVA (*p *< 0.05);

Among the eutrophic children, the higher the consumption of beans, the higher the amount of dietary fiber. The obese children from the first tertile presented low amount of fiber in the diet when compared to other tertiles. In the third tertile, the eutrophic children consumed more fiber than the obese children.

In the eutrophic group, it was possible to observe that the proportion of fat in the diet was higher in the first tertile when compared to the second and third tertiles, the same was not verified in the obese group.

In general, there was a tendency of higher amount of iron as the intake of beans increased. The eutrophic children from the third tertile presented higher amount of iron in the diet when compared to children from other tertiles. The obese children from the third tertile of S. beans had greater amount of iron in the diet when compared to children from the first tertile (table 4).

A weak and negative correlation was found between the proportion of fat and the amount of dietary fiber consumed (r = -0.36; *p = *0.001).

### Micronutrients consumed by the eutrophic and obese children

Regarding the micronutrients, calcium, iron, vitamin C and vitamin A were analyzed. Most micronutrients were properly consumed, regardless of the group. Vitamin A was consumed in higher amount by the obese group (917.2 ± 534.2 mcg) when compared to the eutrophic group (512.6 ± 316.3 mcg) (p = 0.0278). As a consequence, the adequacy of vitamin A intake was higher in the obese group (84%) when compared to the eutrophic group (34%). Calcium consumption, regardless of the group, was below the recommended value of adequate intake (AI), (eutrophic group = 92.7%; obese group = 95%) (Table 1).

## Discussion

The study compared and established differences between nutrient composition and the meal patterns of obese and eutrophic students. Between the groups, there were no differences in total energy and the amount of macronutrients consumed, except for the intake of dietary fiber, which was higher in the eutrophic group, indicating differences in dietary pattern.

Although the benefits of fiber are already a consensus, the role of fiber in the regulation of body weight is not highly valued, focusing more on the effects of energy and fat content in a diet. According to Slavin *et al *(2005) [11], this situation occurs because when the diet is low in fiber, it is usually high in calories and fat, therefore the effects of fiber can be masked. Despite the fact the amount of calories have become the focus of attention, some studies have found that nutritional balance is the predominant factor for obesity prevention [11-13].

Several studies have found associations between dietary fiber and anthropometric measures [14-20]. There are several mechanisms by which dietary fiber may regulate body weight, such as intrinsic effects (chewing and palatability), effects of the colon (production of short chain fatty acids) and hormonal effects (decreased insulin and increased Cholecystokinin) [20,21].

Among these mechanisms, the increase in the intestinal transit was what could have mostly contributed to the regulation of body weight in this present study, considering that high intakes of dietary fiber are associated with increased fecal loss of energy, fat and nitrogen [22]. Also, there were no differences in energy intake between the groups, showing that the fiber did not have any influence in satiety.

Beans were the main food that influenced the amount of dietary fiber. It is believed that dried beans were more consumed because they last longer after the purchase. Besides, they have a historical importance in the Brazilian nutrition, being responsible for nutritional survival of most Brazilians [23].

In addition to the high protein content, beans have high amounts of soluble fiber, soluble vitamins and minerals. Considering these qualities, some countries are specifying the amount of daily servings of dried beans to be consumed by the population [24,25] and studies have confirmed that the intake of beans increases the quality of the diet, [17,26] while decreasing the amount of saturated fat and total fat [26].

In this study, it was determined that the higher the intake of beans, the higher the amount of fiber and iron and the lower the proportion of fat in the diet. This lower proportion of fat was evident in the eutrophic group. In this group, the higher intake of beans may have contributed to the lower intake of other energy-dense food, high in fat.

In the eutrophic group, a better correlation was verified between beans and fiber was verified when compared to the obese group, perhaps because in the third tertile of S. beans the eutrophic group had a higher intake of fiber. In some studies, the consumption of beans has influenced the weight loss of individuals [18,27-29].

It is presumed that the children who did not consume beans replaced a family meal with food that presented low fiber content but similar energy density. Foods that have the same amount of total energy but different amounts of fiber can exert distinct effects on body weight regulation [30].

This substitution can be confirmed by a higher adequacy in the consumption of vitamin A among obese children. It is assumed that this greater amount is related to increased intake of processed foods (e.g., cookies, chocolate powder) whose target are children. They are often enriched with vitamins and minerals, but hide high contents of fat, sugar and sodium.

Regarding the fruits, worrying results were found due to the low consumption. Most students did not consume fruits. The children in this study come from low income families and usually, in this situation, fruits are bought only once a month. The eutrophic group showed stronger correlation between the consumption of fruits and fiber compared to the obese group. This difference may be due to the different types of fruits consumed. In the eutrophic group, apples were more consumed than in the obese group. The pectin, which is the main fiber of this fruit, seems to benefit the weight loss [15].

Also in the eutrophic group, the tangerine was the favorite citrus fruit and according to the Brazilian nutritional database, it has a better source of fiber when compared to orange [31]. In general, calcium intake was below the recommended AI, being this is a reflection of low milk consumption, which has been reported in some studies [9,32].

During the family meal, the intake of rice with beans is traditional, but this dietary pattern is changing. The consumption of beans is decreasing, especially among young people [33]. In this present study, the low consumption of this legume and other vegetables influenced the large inadequacy of fiber intake in the groups. The urbanization favors this decline, once it contributes to the short time to prepare a homemade meal and moreover, the fast foods have more appeal due to the better palatability found in fat and sugar [34,35]. This preference for sweets and fat -energy dense foods over grains, legumes and vegetables is not restricted to Brazilian children; other countries are experiencing the same situation [36,37]. Recent reviews have shown association between skipping meals and an increased overweight and obesity risk in children [38,39], with an emphasis on low consumption of breakfast [39]. Increased meal frequency not only avoids high-fat, energy-dense foods and soft drinks consumption but also reduces postprandial metabolic and endocrine responses due to nutrient consumption, with lower insulin secretion [38,39].

Finally, although the influence of energy intake in weight gain was not verified, underreport and forgetfulness cannot be ignored. Children, especially obese, may be embarrassed to report the "unhealthy" food and children who replace meals with many snacks during the day may not be aware of all the food that is being consumed, underestimating the total energy of the diet. Considering the short time parents had to take part in this study, we had some limitations with the small sample size. However, we believe that this study showed some interesting results that should be investigated with a bigger sample size.

## Conclusions

The results indicate that low intake of dietary fiber is associated with risk for obesity; this consumption was strongly linked to the consumption of servings of beans. This reinforces the importance of traditional family meals, which are being daily replaced with snacks and processed foods. The child's nutrition is an important factor in health, considering that the eating habit formed in childhood can persist throughout the adulthood, influencing the quality of life of the individuals.

## Methods

### Recruitment

This study, derived from a main project titled: "Risk factors for chronic diseases in obese schoolchildren", was developed in a São Paulo inner city with 7 to 11 year-old children enrolled in six elementary public schools. The total sample was 83 children, being 42 obese and 41 eutrophic. The study was submitted to the research ethics committee for approval at Unesp - Araraquara (Protocol Number. 31/2005). Only children whose guardians agreed to and signed a consent form took part in this study.

Children with obesity (≥ 95th percentile for the age- and sex-specific BMI values according to the U.S. Growth Chart 2000) and with eutrophy (percentile for the age - and sex-specific BMI values between 5 and 85) [40] were recruited. In table 5, the anthropometric characteristics of children who participated in the study are described, confirming high values of BMI, waist circumference and body fat percentage in the obese group when compared to the eutrophic group.

**Table 5:** Anthropometric variables and age of the obesity and eutrophic group

Measures	Groups		
	Eutrophic (n = 41)	Obesity (n = 42)	*p-*value*
*Age (Year)*	8.9 ± 1.0	8.9 ± 1.0	0.9209
*BMI (kg/m^2^)*			
90^th^	18.6	29.4	
Mean ± sd	16.4 ± 1.5	25.1 ± 4.0	p < 0.00001
10^th^	14.8	21.1	
*Waist circumference (cm)*			
90^th^	63.0	89.3	
Mean ± sd	57.2 ± 5.0	78.1 ± 8.8	p < 0.00001
10^th^	51.1	66.1	
*Body fat percentage (%)*			
90^th^	29.0	54.3	
Mean ± sd	20.4 ± 6.7	39.9 ± 9.6	p < 0.00001
10^th^	12.0	31.0	

* by student-t test; BMI = body mass index

### Anthropometry

For the anthropometric measures, the schoolchildren were barefoot with minimum clothes. A properly calibrated digital scale, Plenna^® ^brand with a 150 kg capacity was used for weighing. The height was measured with a TBW ^®^stadiometer. Circumference of waist was measured following the protocol of Callaway *et al *(1988) [41]. The percentage of body fat was measured by impedance with a portable foot to foot scale Tanita^® ^TBF 310, this model has been validated to be used in children [42] and in groups of obese children [43].

### Food Intake assessment

Food intake was evaluated through two non-consecutive 24 h food recalls. This method is considered a standard reference and has already been validated in previous studies [44,45]. With the help of an album of photographs and kitchen utensils, the students were asked to report everything that was consumed during the previous 24 hours. The amount of food consumed was recorded in household measures, transformed into grams and then converted into numbers of food servings, according to the energetic equivalence of each food group pyramid.

To estimate the intake of energy and nutrients, foods (in grams) were reported and analyzed by a program of Nutrition (NutiWin^®^) [46].

The percentage distribution of energy among the macronutrients was analyzed according to the cutoff points of the Acceptable Macronutrients Distribution Range - DRIs. When the proportion of energy from protein was lower than 10%, it was considered low intake and above 30%, it was considered as high. When the intake of Carbohydrate was below 45%, it was considered low, when it was above 65%, the consumption was considered high. A high intake of fat was considered above 35%, a low intake was considered below 25% [47].

To assess the adequacy of iron, vitamin C and vitamin A intakes, we used the EAR's (*Estimated Average Requirement*), from DRI's. The EARs can be defined as the average nutrient amount for a given stage of life and gender and it is a value of reference that was used to be compared to the average amount of micronutrients consumed by the groups of children [48,49]. The micronutrient that did not present the EAR value (calcium and fiber) was evaluated by the AI from DRI's. This value does not determine the inadequate intake, but just the recommended intake for healthy people [50]. Then, to estimate the proportion of individuals consuming above or below the EAR, considering the variation of inter-and intra-personal consumption, we applied a statistical method to remove day-to-day variability, reflecting only the change in consumption between individuals from the group [51].

### Statistical Analysis

For statistics, we used Microsoft Excel (2003) and Bioestat 5 programs. Differences in group averages were analyzed by student-t test. Anova one criterion was used to test differences in tertile averages. The relation between data was tested by Pearson correlation. The correlation coefficients were classified as perfect (= 1.00), strong (> 0.75), medium (> 0.5), weak (< 0.5), and null (= 0.00). Differences among proportions of nominal data were tested by chi-square (χ^2^) and by Yates' chi-square test when necessary. The level of significance was 5% in all comparisons. The normality of the data was analyzed with Lilliefors test.

## Competing interests

The authors declare that they have no competing interests.

## Authors' contributions

EAB and MRMO designed and initiated the study. EAB performed the statistical analyses. All authors helped gather and interpret data and write the article. All authors approved the final version of the article. The study was submitted for approval by the ethics committee research form the faculty of Pharmaceutical Sciences of Unesp - Araraquara (protocol No. 31/2005). There are no conflicting interests regarding the funding of the study.
